# Integrating Geriatric Assessment into Decision-Making after Prostatectomy: Adjuvant Radiotherapy, Salvage Radiotherapy, or None?

**DOI:** 10.3389/fonc.2015.00227

**Published:** 2015-10-15

**Authors:** Aurore Goineau, Bénédicte d’Aillières, Laure de Decker, Stéphane Supiot

**Affiliations:** ^1^Radiation Oncology, Institut de Cancérologie de l’Ouest Papin, Angers, France; ^2^Institut de Cancérologie de l’Ouest Papin, Angers, France; ^3^Institut de Cancérologie de l’Ouest René Gauducheau, St Herblain, France; ^4^Radiation Oncology, Institut de Cancérologie de l’Ouest René Gauducheau, St Herblain, France

**Keywords:** post-operative radiotherapy, prostate cancer, elderly patients, geriatric assessment, adjuvant radiotherapy, salvage radiotherapy

## Abstract

Despite current advancements in the field, management of older prostate cancer patients still remains a big challenge for Geriatric Oncology. The International Society of Geriatric Oncology (ISGO) has recently updated its recommendations in this area, and these have been widely adopted, notably by the European Association of Urology. This article outlines the principles that should be observed in the management of elderly patients who have recently undergone prostatectomy for malignancy or with a biochemical relapse following prostatectomy. Further therapeutic intervention should not be considered in those patients who are classified as frail in the geriatric assessment. In patients presenting better health conditions, salvage radiotherapy is to be preferred to adjuvant radiotherapy, which is only indicated in certain exceptional cases. Radiotherapy of the operative bed presents a higher risk to the elderly. Additionally, hormone therapy clearly shows higher side effects in older patients and therefore it should not be administered to asymptomatic patients. We propose a decision tree based on the ISGO recommendations, with specific modifications for patients in biochemical relapse.

## Introduction

Prostate cancer is the most common form of cancer in European and American men, particularly afflicting older patients by missing the target of an effective therapy quite often toward under-treatment (1–5). However, two large studies of non-curative approaches to prostate cancer have demonstrated, independently of age, that patients at low and intermediate risk have a lower specific mortality when compared to high-risk patients (64%) (6, 7) and therefore professional bodies are not following these directories (8, 9). Indeed, the importance of patient’s age is going to be considered, as reported by the recent recommendations of the European Association of Urology (EAU) (10). In the meantime, the International Society of Geriatric Oncology (ISGO) piloted a multi-disciplinary working group (of urologists, radiotherapists, medical oncologists, and geriatricians), delivering a set of guidelines for the treatment of prostate cancer-affected elderly patients (based on the available literature), which were updated in 2013 (11, 12).

However, while this guidance addresses both localized prostate cancers and metastatic disease, the question of post-operative radiotherapy was not addressed, nor the problem of biochemical relapse, though these are frequent scenarios in elderly patients. We propose here a revision of the model that addresses this particular situation.

## Wait and See Policy and Best Supportive Care

The first question in a clinician’s mind when confronted with a patient presenting with high-risk pT3 prostate cancer with positive margins, detectable post-operative PSA levels, or a biochemical relapse, is the importance and relevance of all treatments, whatever they might be. The life expectancy of a patient at the time of biochemical relapse can be considerable (13). PSA doubling time and the initial Gleason score are the variables that are currently considered the best predictors of prostate cancer-specific mortality (14–16). In an illustrative study, Antonarakis followed a series of 450 patients in biochemical relapse, observing a metastasis-free survival rate (MFS) at 10 years of 94% (Gleason score 4–6) and 19% (Gleason score 8–10). In those cases where the PSA doubling time (PSA DT) was more than 15 months, the MFS rate reached the 72%, in contrast to those where the PSA DT was less than 9 months (7% only) (17). Indeed, tumor aggressiveness and specific mortality risks should be discussed between urologists and radiation oncologists and integrated with a general onco-geriatric opinion regarding patient’s conditions or any existing co-morbidity (as specifically recommended by the new paragraph in the EAU guidance). Therefore, further therapeutic interventions should be considered only in patients presenting an aggressive prostate cancer (Gleason score ≥8 and/or short PSA DT), in accordance with a geriatric opinion. If any decision will be taken at this regard, the choice of therapy and its timing is the next consideration.

## Adjuvant or Early Salvage Radiotherapy for Older Patients

The rationale for post-operative irradiation is addressed to eradicating any microscopic residual of tumor after prostatectomy. In case of a detectable disease (a treatable PSA level), such therapy is termed “salvage radiotherapy (SRT),” while in cases where there are concerns about the completeness of the surgical resection, either positive excision margins or capsular rupture, though with sub-treatable PSA levels, the therapy would be termed “adjuvant radiotherapy” (ART).

For high-risk prostate cancers (pT3, R1), three large trials have validated the use of ART with respect to follow-up in terms of survival with no biochemical relapse: EORTC 22911, ARO 9602, and SWOG 8794 [reviewed in Thoms et al. (18–21)]. In the EORTC trial, 5-year survival without biochemical relapse was 77% in the ART arm against 55% in controls (which included patients who had late SRT). However, the survival data from these trials in patients with distant metastases are inconsistent. Of the three trials, only the EORTC trial specifically analyzed the data with respect to age, though patients older than 75 years were excluded from the trial. Patients over 70 years nevertheless represented 20% of all patients recruited (196/1,105 of whom 94 patients were in the radiotherapy arm and 102 in the control arm), against the 47% who were under 65 years old. It is important to note that it was only in this over-70 patient group that survival without biochemical relapse was not improved by adjuvant treatment compared with watchful waiting. This study also showed that ART clearly led to worse outcomes for the over-70 group in terms of survival free from clinical relapse [HR = 1.78 (1.14–2.78), *p* = 0.0003] and overall survival [HR = 2.94 (1.75–4.93), *p* = 0.0008]. The criterion of age was the only significant predictor among the many survival variables studied, which included PSA level, resection margins, extra-capsular invasion, invasion into the seminal vesicles, and pT staging.

Three multi-center randomized controlled trials are currently underway (GETUG 17, RAVES, and RADICALS), comparing immediate ART with radiotherapy according to biochemical parameters, with no upper age limit for study inclusion. Until these trials report (which will not be for several further years), the current recommendation for elderly patients is to refrain from ART, but to consider SRT early, should the PSA rise above 0.2 ng/ml, whereas younger patients may benefit more from ART (20, 22).

## Salvage Radiotherapy: Effectiveness and Adverse Effects

Salvage radiotherapy is the only potentially curative treatment available in biochemical relapse. Early SRT is thought to prevent tumor progression in around half of patients (23). In the study by Stephenson, 501 patients (between 40 and 79 years at the time of their prostatectomy procedure) were given SRT, and 50% were relapse-free at 4 years. For patients with progressive disease, the median time to progression (TTP) was 12.5 months. It should be noted that this analysis does not take account of the age of the patients. This author has also developed a predictive model of relapse-free survival (biochemical or clinical) within 6 years of SRT (15). The model was developed using a retrospective series of 1,540 patients between 58 and 67 years at the time of prostatectomy (though with no age data at the time of irradiation). The relevant variables in this nomogram are: PSA level before SRT, Gleason score, PSA DT, surgical margins, lymph node status, and the administration of hormonal treatment before or after SRT. This nomogram, which is suitable for patients of all ages, may assist decision-making when the PSA level is rising.

Radiotherapy to the prostatic bed can have long-term adverse effects. In the EORTC study, late grade 3 complications (from all sources) occurred in 5.3% of cases (compared with 2.5% in the observation arm, *p* = 0.052) with all genitourinary toxicity of grade ≥2 at 21% (compared with 13% in the observation arm, *p* = 0.003), though no significant difference in gastro-intestinal toxicity of grade ≥2 were observed (2.5 versus 1.9%, *p* = 0.47) (18). These potential late complications are mainly urethral stenosis, urinary incontinence, and rectal bleeding. They were not specifically analyzed with respect to patient age. However, there are reasons to believe that SRT leads to fewer late side effects than ART. In a large multi-center retrospective study of 959 patients treated with radiotherapy to the prostatic bed, ART independently predicted late urinary toxicity of grade 2 or greater compared with SRT (24). This study reminds us of the importance of a delay between prostatectomy and irradiation to maximize sphincter recovery, recommending an interval of at least 2 years in order to minimize the risk of late complications. It seems that post-operative radiotherapy leads to a greater number of adverse effects in elderly patients compared with their younger counterparts (25–27) (see Table 1). In another retrospective study of 742 patients, the age and the dose of radiation were the most relevant parameters for predicting grade 3 urinary toxicity in the long term (8 years) (25). The mean age at the time of radiotherapy was 65 years, with 117 patients less than 72 years and 69 patients aged over 71 years. Grade 3 urinary toxicity occurred in 16% of patients aged over 71 years, in comparison with 6% aged less than 72 years (*p* = 0.006). In a multivariate analysis, age was in independent prognostic predictor of long-term grade 3 urinary toxicity, with an HR of 4.26 (1.45–12.47), *p* = 0.004.

**Table 1:** **Post operative radiotherapy adverse events according to age**.

Reference	N	Adverse event studied	cut off (years)	Hazard ratio/odds ratio
Cozzarini et al. (2012) (25)	742	G3 long-term GU complications	71	HR = 4.26 (1.45–12.47), *p* = 0.004
Longobardi et al. (2011) (26)2011	178	≥G2 acute bowel complications	66	OR = 4 (0.9–18.6), *p* = 0.08*
Perna et al. (2010) (27)	96	≥G2 acute bowel complications	Continuous	OR = 1.13 (1.02–1.25), *p* = 0.021

*This study designated p < 0.1 as significant.GU: Genito-urinary

We have not yet raised the question of hypofractionated treatment. In prostate cancer, and particularly in the elderly patient, increasing the dose of radiation in each fraction, whilst reducing the number of sessions, is an attractive concept. Retrospective studies have evaluated the potential risks of increased toxicity associated with hypofractionation and studies are under way to evaluate its effectiveness and the potential risks of increased toxicity associated with hypofractionation (28, 29).

## Androgen-Deprivation Therapy

Patients presenting a localized prostate cancer who are currently considered ineligible for a curative local therapy (though most often the radiotherapist or urologist uses “intuitive criteria” to make this decision) are often offered androgen-deprivation therapy (ADT) instead. Scientifically, there is no evidence of benefit in survival to giving early treatment (30, 31). It is therefore currently advised to treat these patients only if they become symptomatic, except for patients who present with rapidly progressive disease (PSA DT <12 months). However, this evidence is balanced in practice by the concerns of patients, who, knowing that their PSA is climbing, are often very demanding that some treatment have to be instituted. It is important to note that very few patients are then referred for an onco-geriatric assessment, and that local treatment is judged more hazardous than hormone therapy. However, the long-term adverse effects of hormone therapy are now well-recognized and of particular concern in the elderly (32). Such adverse effects include bone demineralization (33), increased fracture risk (34, 35), and increased cardio-vascular risk (36, 37). Several studies have found that patients rapidly decline physically, with marked effect on the quality of life, when treated with hormone therapy (38). Numerous physical activity programs have been devised to limit this, with very encouraging results (39–41). The other option to improve tolerability is to give intermittent hormone therapy rather than continuous treatment (42). This therapeutic strategy has been found to be equivalently effective, and is associated with a reduction in the unwanted effects of hormone therapy in several trials, notably in one of the largest trial, that of Calais da Silva, which recruited more than 900 patients and was also confirmed in a meta-analysis published by Shaw (43, 44).

It is also important to underline that, in practice, brief hormone therapy can be used alongside SRT. In high-risk localized cancers, a combination of radiotherapy and hormone therapy has generally been found to be more effective in comparison with radiotherapy alone (45). Among these trials, it should be noted that the 85.31 trial organized by the RTOG, included patients whose pT3a or b stage disease had been operated on, representing around 15% of the total number of 977 patients recruited to the study (46). The authors of this trial also concluded that combined radiotherapy and hormone therapy was superior, both in terms of overall survival (39 versus 49%, *p* = 0.002) and disease-specific mortality (16 versus 22%, *p* = 0.005). Similarly, post-operative radiotherapy combined with ADT may represent the new standard in the near future, based on the results of different clinical trials such as RTOG 9601, RTOG 0534, GETUG 16, and GETUG 22 trials (47). However, the risk of cumulative toxicity following the two treatments has to be considered. Mature results of these different trials are needed prior to concluding that all biochemically relapsing prostate cancer patients need to be treated with prostate bed radiotherapy and 6-month ADT.

## Geriatric Assessment and ISGO Guidelines

It is currently considered that a patient will benefit from local treatment for his prostate disease if his life expectancy exceeds 10 years. But life expectancy is not only determined by age. This is why it is fundamentally necessary to conduct an evaluation that takes into consideration co-morbidities, independent living, nutritional status, cognitive function, and other important predictors of death not linked to the cancer of the elderly patient in localized prostate cancer, before making treatment decisions. Among the multi-dimensional geriatric evaluations used in onco-geriatrics, several tools and scoring systems have been developed. The burden of co-morbidities can be assessed using the Charlson score, or preferably, the Cumulative Illness Score Rating-Geriatrics (CIRS-G) (48). In that study of 2,273 patients whose prostate cancer was treated with the objective of cure, a CIRS-G score of 1 translated into a relative risk of death within 10 years from another cause than prostate cancer of 1.64 (1.52–1.76), when compared with a CIRS-G score of 0. The relative risk rose by 1.18 (1.15–1.21) with each additional point gained using the scoring system. Independent living is assessed using the activities of daily living (ADL) and instrumental activities of daily living (IADL) scores (49, 50). In a study of 9,467 men and women over 70 years old, the survival rate at 10 years was 54.2% in ADL score 0 patients (fully independent), versus 31.3, 22.5, 16.7, and 4.2%, respectively, for ADL score groups 1–4 (reflecting increasing dependence), differences which were all statistically significant (50). Nutritional status may be evaluated by using body weight, the rate of loss of body mass, and the Mini Nutritional Assessment (MNA) (51). For a MNA <17, the risk of death within a year is 50%, while that of patients with an MNA score between 17 and 23.5 is halved, at 25%. It is also important to evaluate cognitive functions and patients behavior systematically.

The onco-geriatric assessment enables the development of an accurate picture, which represents the patient’s overall condition, enabling appropriate interventions to be instituted where possible, such as the provision of help at home, the introduction of an anti-depressant, dietetic advice, and modification of the home environment. The outcome of this assessment is to place patients in one of three groups: fit, vulnerable (with potentially ameliorable conditions), and frail (whose condition is irreversible) (52). Such thorough assessment is extremely time-consuming, and may not be possible in routine practice for every elderly patient who presents with prostate cancer. The G8, a geriatric rating instrument with only eight questions yielding up to 17 points, has been developed for screening in this situation (Table 2). Patients who score 14 or more are fit, and should be treated similarly to younger patients. Patients scoring ≤14 should ideally be referred for complete onco-geriatric assessment (53, 54). The usefulness of systematic onco-geriatric assessment has largely been demonstrated, by improving the patient’s overall condition, or by informing therapeutic decision-making (55–57). The International Society of Geriatric Oncology (SIOG) convened a multi-disciplinary working group of urologists, radiotherapists, medical oncologists, and geriatricians charged with reviewing the literature and produced a set of guidelines on the treatment of prostate cancer in elderly patients (11, 12). These guidelines were then adopted by the EAU in its specific section on the elderly patient. However, prostate cancers are dealt with in two categories depending on whether the disease presents with localized or metastatic disease. The guidelines do not specifically consider the problem of biochemical relapse, though it is a frequently encountered situation in routine clinical practice.

**Table 2:** **ONCODAGE scoring chart for establishing G8 score**.

**Has the patient lost his appetite? Has he eaten less in the last 3 months because of poor appetite, gastro-intestinal symptoms, dysphagia, or problems with mastication?**
0: Severe anorexia
1: Moderate anorexia
2: No anorexia
**Recent weight loss (in the last 3 months)**
0: Weight loss >3 kg
1: Not known
2: Weight loss >1 kg and <3 kg
3: No weight loss
**Mobility**
0: Bed-bound or wheelchair-bound
1: Mobile within the home
2: Independently mobile
**Neuropsychological problems**
0: Severe dementia or depression
1: Moderate dementia or depression
2: No psychological problem
**Body mass index (BMI)**
0: BMI < 18.5
1: 18.5 ≤ BMI < 21
2: 21 ≤ BMI < 23
3: BMI ≥ 23
**Taking more than three drugs**
0: Yes
1: No
**Does the patient consider his own health too be better or worse than others of his own age?**
0: Less good
0.5: Don’t know
1: As good as others
2: Better than others
**Age (years)**
0: >85
1: 80–85
2: <80
**Total 0–17**

**Figure 1:**
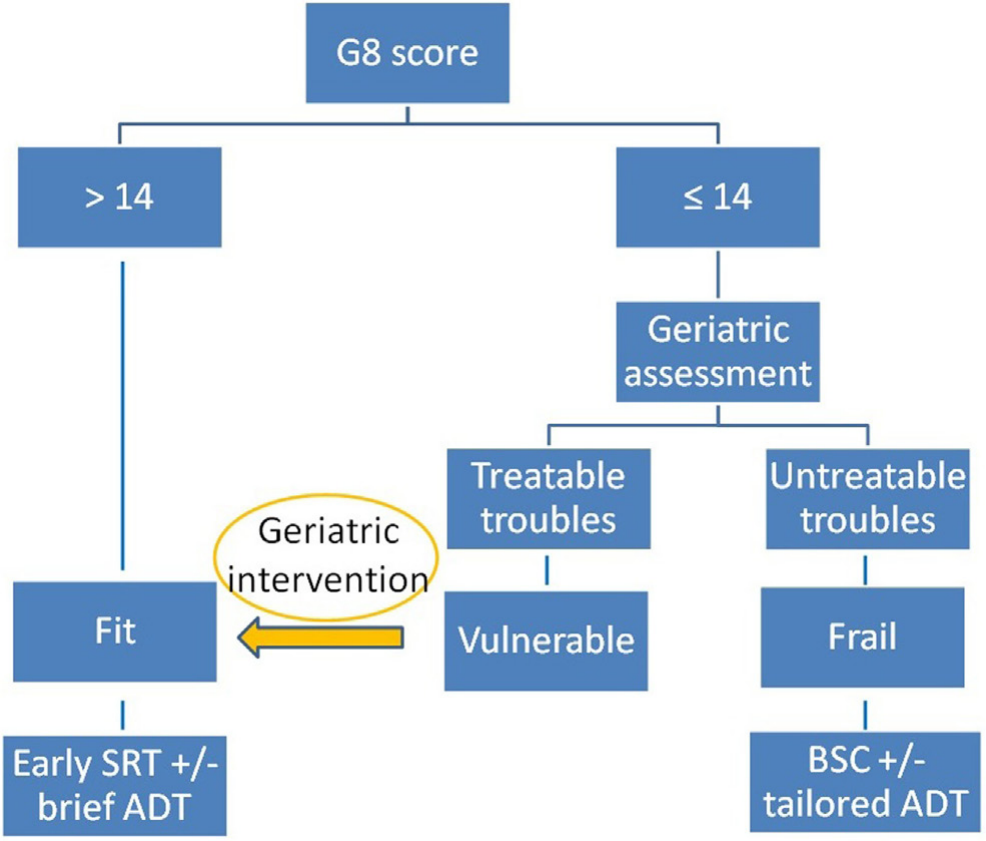
**SRT: Salvage radiotherapy; ADT: androgen deprivation therapy; BSC: best supportive care**.

In the case of biochemical relapse following prostatectomy in patients over 70 years of age, we propose that the G8 screening questionnaire should be administered by the urologist, radiotherapist, or medical oncologist (Figure 1). If the G8 score is >14, the patient is considered fit and will be preferentially offered SRT if his PSA >0.2 ng/ml. (ART may be considered on a case by case basis in particularly aggressive disease). If the G8 score ≤14, the patient will be referred to the onco-geriatric service for complete assessment. If this finds the patient to be vulnerable or frail, any treatable conditions should be addressed in order that the patient may benefit from SRT. If the patient is considered to be frail or unfit, with irreversible decline, supportive care should be offered, and hormone therapy delayed as long as possible, to be used only in the advent of bony or urinary symptomatology.

## Conclusion

Appropriate assessment and management of elderly patients with prostate cancer is a key issue. The appropriate balance between the risk of under-treatment (on the grounds of age alone), and the risk of adverse effects that may excessively compromise the patient’s general status and independence, must be determined for each patient. In practice, in the post-operative situation in the elderly patient, the following principles may be adopted:

- No ART except in exceptional cases, while favoring SRT.
- Radiotherapy of the prostate bed presents higher risk in the elderly patient compared with his younger counterpart.
- Hormone therapy as a monotherapy is clearly toxic to elderly patients, and should not be given in the absence of symptoms.
- Short-term Hormone therapy combined with salvage prostate bed radiotherapy may represent a new standard treatment in the near future, but more mature data from clinical trials are needed.

Onco-geriatrics has been a growth specialty for several years now, and professional bodies increasingly provide guidance specific to the needs of older patients. Nevertheless further onco-geriatric trials remain necessary with the aim of establishing the place of more aggressive treatments such as SRT or hormone therapy in the treatment of elderly patients, compared with watchful waiting.

## Conflict of Interest Statement

The authors declare that the research was conducted in the absence of any commercial or financial relationships that could be construed as a potential conflict of interest.
